# Diabetic Kidney Disease: From Pathophysiology to Regression of Albuminuria and Kidney Damage: Is It Possible?

**DOI:** 10.3390/ijms26178224

**Published:** 2025-08-24

**Authors:** Georgia Doumani, Panagiotis Theofilis, Aikaterini Vordoni, Vasileios Thymis, George Liapis, Despina Smirloglou, Rigas G. Kalaitzidis

**Affiliations:** 1 Center for Nephrology “G. Papadakis”, General Hospital of Nikaia-Piraeus “Ag. Panteleimon”, 18454 Nikaia, Greece; geo.doumani@gmail.com (G.D.); panos.theofilis@hotmail.com (P.T.); katerinavord@gmail.com (A.V.); vthimis@gmail.com (V.T.); desmirlo@yahoo.com (D.S.); 2 1st Department of Pathology Medical School, National and Kapodistrian University of Athens, Laikon Hospital, 11627 Athens, Greece; gliapis@gmail.com

**Keywords:** type 2 diabetes mellitus, chronic kidney disease, albuminuria, renin-angiotensin-aldosterone system inhibitors, SGLT2 inhibitors, glucagon-like peptide 1 receptor agonists, mineralocorticoid receptor antagonists, aldosterone synthase inhibitors, combination treatment

## Abstract

Diabetes mellitus (DM) poses an increasingly high global health burden nowadays, while in adults, chronic kidney disease (CKD) associated with DM impacts 20–40% of those with the condition. Effective management of CKD in patients with diabetes necessitates a comprehensive, multidisciplinary approach. Numerous factors, including glomerular hyperfiltration, oxidative stress, inflammation, and hypoxia are linked to the advancement of diabetic kidney disease (DKD). Currently, no specific treatment for DKD has been established, prompting extensive exploration of new approaches. Renin-angiotensin-aldosterone system inhibitors and sodium-glucose cotransporter 2 inhibitors have demonstrated renoprotective effects in various human clinical trials. Additionally, glucagon-like peptide 1 receptor agonists and mineralocorticoid receptor antagonists have been reported as effective in managing DKD, while new therapeutic candidates are also under investigation, such as soluble guanylate cyclase activators and aldosterone synthase inhibitors. Recent evidence has shown that treating diabetic nephropathy by reducing albuminuria levels and retarding its progression is a complex skill. The purpose of this review is to support the impressive results that appear in reducing albuminuria and the progression of diabetic nephropathy with early and intensive combination treatment compared to the recently emerged conventional monotherapy, with agents that act on different pathophysiological mechanisms.

## 1. Introduction

Diabetes mellitus (DM) poses an increasingly high global health burden nowadays. It encompasses a collection of metabolic disorders related to carbohydrate metabolism, characterized by the inadequate utilization of glucose as an energy source and its excessive production stemming from improper gluconeogenesis and glycogenolysis, leading to hyperglycemia. DM is clinically classified into several types: type 1 diabetes mellitus (T1DM), type 2 diabetes mellitus (T2DM), gestational diabetes, and less common forms caused by specific genetic defects, pancreatic exocrine disorders, or medication effects [1]. On the other hand, chronic kidney disease (CKD) is characterized by a persistent rise in urinary albumin excretion (albuminuria), a diminished estimated glomerular filtration rate (eGFR), or other indicators of renal dysfunction [2].

In adults, CKD associated with DM impacts 20–40% of those with the condition [3]. Diabetic kidney disease (DKD) manifests in individuals with DM and diminished kidney function, which may arise from various causes, such as hypertensive nephrosclerosis and persistent acute kidney failure. Diabetic nephropathy (DN) is a term used to describe pathological structural and functional alterations observed in the kidneys of patients with DM that are attributable to the impact of DM on renal health. These alterations lead to certain clinical presentations. The hallmark indicator of DKD is albuminuria, which correlates with the advancement of renal disease and cardiovascular events. Multiple mechanisms—altered renal hemodynamics, oxidative stress, inflammatory pathways, hypoxia, and excessive renin–angiotensin–aldosterone system (RAAS) activation—contribute to DKD pathogenesis, with renal fibrosis serving as a pivotal end-stage feature [4]. Regression in medicine refers to the partial or complete reversal of a disease’s signs and symptoms. In DKD, regression can be defined as a change from a higher category of albuminuria to a lower category—e.g., from A3 to A2, or from A2 to A1—which represents a slower decline in kidney function. This change can be used to monitor disease progression. Another approach involves the histological evaluation of renal biopsy specimens, which reveals the severity of kidney damage; however, this method is not feasible in a large number of patients. A different clinical course of DKD, apart from the above, also has been reported, with patients showing declined kidney function with normal levels of albuminuria, known as the ‘non-albuminuric DKD [5].

Effective management of CKD in patients with diabetes necessitates a comprehensive, multidisciplinary approach. Those with diabetes and CKD face heightened risks for acute complications related to diabetes, including hypoglycemia and diabetic ketoacidosis, as well as long-term issues including retinopathy and neuropathy. There is also an increased likelihood of kidney failure requiring dialysis or transplantation, along with significant cardiovascular risks, such as arrhythmia, ischemia, myocardial infarction, and heart failure. Thus, thorough diabetes management must encompass regular assessments for these complications and address various cardiovascular risk factors beyond hyperglycemia, included in the recently defined cardio-kidney-metabolic syndrome [6], namely, hypertension, dyslipidemia, obesity, and lifestyle considerations [2,3,7].

Given the complexity and heterogeneity of DKD, it remains uncertain whether current interventions can fully halt or reverse its progression. This review aims to assess the evolving understanding of DKD pathophysiology and explore whether complete regression of kidney damage is a realistic therapeutic goal in the era of pharmacological milestones.

## 2. Pathophysiology of DN

**Figure 1:**
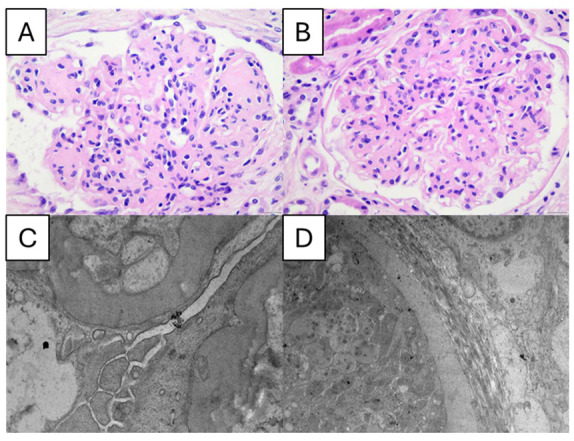
Pathological features of diabetic nephropathy. (**A**) Mesangial nodules in a severely enlarged glomerulus, in association with thickening of glomerular basement membranes (GBM) and microaneurysmal dilatation of GBM, corresponding to a case of diabetic nephropathy class III (nodular sclerosis, Kimmelstiel–Wilson nodule/lesion), according to classification (H&E × 400). (**B**) Diffuse severe mesangial matrix expansion, in association with thickening of glomerular basement membranes, corresponding to a case of diabetic nephropathy, class IIb (severe mesangial expansion) according to classification (H&E × 400). (**C**) Severe thickening of glomerular basement membranes (Uranyl acetate × 12.000, electron microscopy). (**D**) Severe thickening of tubular basement membranes (Uranyl acetate × 5.000, electron microscopy).

The progression from diabetes to DN and ultimately to end-stage renal disease (ESRD) is driven by a complex interplay of metabolic and hemodynamic disturbances. Chronic hyperglycemia fosters the accumulation of advanced glycation end products, stimulates growth factor release, and disrupts hormonal and vascular regulation. These changes promote oxidative stress and inflammatory activity, which in turn lead to glomerular hyperfiltration, elevated intraglomerular pressure, and kidney tissue hypertrophy. Over time, these processes alter the cellular and extracellular composition of renal structures, producing the clinical hallmarks of albuminuria and hypertension [8]. Pathologically, the earliest detectable change—often occurring within a few years of diabetes onset—is thickening of the glomerular basement membrane. This abnormality is paralleled by thickening of tubular and capillary basement membranes. As the disease advances, additional changes emerge, including podocyte depletion with loss of foot processes, expansion of the mesangial matrix, and loss of endothelial fenestrations [2,9,10]. Later, mesangial volume continues to expand [7,8,10,11]. Segmental mesangiolysis is seen when diabetes worsens and is believed to be linked to the emergence of microaneurysms and Kimmelstiel/Wilson nodules, which frequently coexist. Exudative lesions are caused by subendothelial deposits of plasma proteins, which build up in microaneurysms, arterioles, glomerular capillaries, and small artery branches. These deposits are periodic, electron-dense, and acid-Schiff positive [2,10,12]. Luminal compromise, such as hyaline arteriosclerosis, may be the consequence of these deposits. Bowman’s capsule (capsular drop lesion) and the proximal renal tubules have comparable subepithelial deposits. Interstitial alterations and glomerulopathy combine to form segmental and global sclerosis in the later stages of diabetes (Figure 1).

Current consensus classifications divide DN into four main categories of glomerular lesions, assessed alongside the extent of interstitial inflammation/fibrosis and vascular changes. The degree of mesangial expansion is strongly associated with renal functional decline, albuminuria, and elevated blood pressure, whereas glomerular basement membrane thickening is less tightly correlated but still considered a marker of disease activity (Table 1).

## 3. Diagnosis of DN

Determining whether a patient has DN or another kidney disease must be the main focus of treatment for DM patients who exhibit signs of renal disease, such as albuminuria, hematuria, or decreased eGFR [2]. Diagnosing DN in patients with T1DM can be made much easier by using the natural history and progression timeline. DN is less likely to occur if considerable albuminuria develops before five years of T1DM or after twenty-five years. Furthermore, the lack of retinopathy may suggest a diagnosis other than DN because 95% of people with T1DM and DN also have diabetic retinopathy [10]. Unfortunately, these epidemiologic hints are less useful for individuals with T2DM, making them more difficult to manage. Since diabetic retinopathy and DN are only associated in roughly 60% to 65% of cases, its absence does not significantly increase the likelihood that DN will be diagnosed. Additionally, the natural history timeline is not as reliable for aiding in diagnosis because the beginning of T2DM is typically unclear. Therefore, it is the responsibility of the practicing doctor to determine whether renal damage is caused by something other than DM. To ascertain if a kidney biopsy would be beneficial, this evaluation usually entails a comprehensive history and physical examination as well as specific laboratory and imaging tests.

Clinically, DKD is defined as a sustained decrease in eGFR < 60 mL/min per 1.73 m^2^ and/or a persistently high urinary albumin-to-creatinine ratio (UACR) ≥ 30 mg/g [2]. DKD screening should be done every year for all T2DM patients starting at the time of diagnosis and every year for patients with T1DM starting five years following diagnosis [8,11]. Regarding the timing of kidney biopsy in DM patients, there are no official guidelines. According to prospective kidney biopsy studies, another diagnosis that would significantly change treatment is unlikely to be detected if a patient with DM has retinopathy (T1DM), proteinuria that appears in the typical timeframe (T1DM), and no signs of another disease (T1/T2DM). Thus, the majority of patients with DM who also have impaired kidney function do not have kidney biopsies [2,7,10].

## 4. Comprehensive Management of DN

Individuals with both diabetes and chronic kidney disease (CKD) frequently live with multiple comorbidities, which heightens their risk for cardiovascular complications, premature death, and progressive loss of renal function. Contemporary recommendations from bodies such as the American Diabetes Association (ADA) [14] and Kidney Disease: Improving Global Outcomes (KDIGO) [15] emphasize a patient-centered, multidisciplinary model of care. This approach integrates structured patient education, active self-management, shared decision-making, and close coordination among specialties to address both metabolic and cardiorenal targets. Optimal care extends beyond glucose control and includes interventions to manage blood pressure, dyslipidemia, body weight, and lifestyle factors. Such measures aim to prevent or delay CKD progression, atherosclerotic cardiovascular disease, and heart failure. Lifestyle optimization—covering dietary patterns, physical activity, and avoidance of tobacco—forms the foundation, while pharmacologic therapies are tailored to preserve organ function and mitigate risk [7,12].

Because many patients require more than one pharmacologic intervention, avoiding therapeutic inertia is critical. Even with treatment, substantial residual risk remains, underscoring the importance of timely initiation and adjustment of therapies. Commonly used agents—such as RAAS inhibitors, sodium–glucose cotransporter-2 inhibitors (SGLT2i), and non-steroidal mineralocorticoid receptor antagonists (ns-MRAs)—can produce early, transient declines in eGFR due to hemodynamic effects. To maximize benefit and minimize adverse events, these drugs are often introduced in sequence, with monitoring to guide titration.

Rapid implementation of evidence-based combinations is facilitated by patient empowerment and coordinated, interdisciplinary follow-up. This proactive, holistic strategy aims to slow renal deterioration, reduce cardiovascular events, and improve quality of life for those with diabetic nephropathy [7,16].

## 5. Treatment of DN

DM was thought to be a kidney illness until the early 19th century, when wasting and increased urine volume were among its symptoms. It was not identified as a metabolic condition until the late 18th century, when glucose was found in blood and urine. DN became increasingly noticeable after insulin became available in the early 1920s, increasing survival. Paul Kimmelstiel and Clifford Wilson’s now-famous 1935 publication, which detailed nodular renal lesions in just 8 maturity-onset persons with diabetes, followed a few isolated accounts that were disregarded [17]. The typical survival period for DN was 5–7 years, and there was no specific treatment available until the late 1970s. It was shown in the early 1980s that lowering blood pressure had kidney-protective effects. Angiotensin converting enzyme inhibitors (ACE-i) were shown to have a superior reno-protective impact on T1DM ten years later [14]. The median survival period from the onset of DN tripled as a result of these advancements in treatment. Angiotensin II receptor blockers (ARBs) showed comparable positive effects on a composite renal endpoint, including death, in two sizable randomized controlled trials conducted in 2001 [18]. Our approach to managing DN has undergone a paradigm shift in recent years. This entails finding numerous new treatment targets and using a “multi-pronged” strategy and the “DKD fantastic four” to help the patient reach a desired renal outcome. This goes beyond controlling blood pressure by inhibiting the RAAS or treating metabolic disorders (as indicated by glycemic control). These trials also show a decrease in cardiovascular risk in addition to renal protection [17].

## 6. RAAS Inhibition in DN

The renin–angiotensin–aldosterone system (RAAS) plays a pivotal role in regulating vascular tone, fluid balance, and organ perfusion. In DN, chronic overactivation of this pathway contributes to progressive structural and functional kidney injury [18,19]. Suppression of RAAS signaling has repeatedly been shown to slow disease progression, with benefits that extend beyond simple blood pressure reduction. Post hoc analysis indicates that the effectiveness of BENEDICT (the Bergamo Nephrologic Diabetes Complications Trial) in reducing albuminuria development was not contingent on lowering blood pressure [15]. Additionally, the main analysis of the ROADMAP (Randomized Olmesartan and Diabetes Microalbuminuria Prevention) experiment revealed that Olmesartan either averted or postponed the beginning of microalbuminuria, with microalbuminuria developing in 8.2% of individuals as opposed to 9.8% of those who received a placebo [20]. RAAS inhibition may therefore protect T2DM patients from developing microalbuminuria [10]. When microalbuminuria progresses to proteinuria, the timetable moves on to the next phase. In the IRMA-2 (Effect of Irebesartan in the Development of Diabetic Nephropathy in Patients with T2DM) study, the potential of irbesartan treatment in patients with T2DM to avoid the incidence of proteinuria was examined. Irbesartan decreased the overall intention-to-treat group’s risk of developing overt proteinuria, which was defined as albumin excretion > 200 mg/d. A dose-dependent benefit was proposed after looking at the subgroups [21].

The impact of two ARBs (irbesartan and losartan) on the development of DN in patients with T2DM, overt proteinuria, and renal dysfunction was examined in the IDNT (Irbesartan Diabetic Nephropathy Trial) and RENAAL (The Reduction in End Points in NIDDM with the Angiotensin II Antagonist Losartan) studies (Table 2). Compared to amlodipine or placebo, irbesartan decreased the risk for the composite endpoint in the IDNT study, which was conducted independently of blood pressure control. Losartan, 100 mg per day, was shown in the RENAAL study to be more effective than placebo in lowering the risk for the same endpoint as in IDNT. The strong evidence offered for the ability of RAAS-blocking drugs to reduce the progression of DN, regardless of blood pressure control [22,23].

There is still a lot of potential for additional therapy and pharmacological research to yield even more benefits, despite the substantial benefit shown with ARBs. In patients receiving ARBs, a decrease in proteinuria is a strong predictor and correlates with the preservation of kidney function. However, not all participants who saw a decrease in proteinuria also experienced a preservation of renal function, and those who benefited did not experience a drop in proteinuria. RAAS-blocking medications are recommended for the treatment of DN patients according to the available evidence. RAAS blockade with multiple agents may be useful in lowering proteinuria, but lacks benefit in preventing ESRD and may be associated with a deleterious adverse event profile, thus prohibiting its widespread use in the treatment of DN [10].

## 7. SGLT2i in DN

Oral hypoglycemic medications, known as sodium-glucose cotransporter 2 inhib-itors (SGLT2i), decrease renal glucose absorption, which raises urine glucose excretion and lowers hyperglycemia [24]. About 97% of the reabsorption of filtered glucose is carried out by high-capacity, low-affinity SGLT2 transporters in the kidney’s proximal tubules, which reduces glycosuria in normoglycemic circumstances [25]. This distinct drug class also has beneficial effects on body weight and blood pressure [26,27,28,29]. The proximal tubules’ increased SGLT2 expression during hyperglycemic episodes raises the glycosuria threshold in diabetic patients [30]. By reducing the renal tubules’ ability to reabsorb glucose by at least 50%, pharmacological inhibition of SGLT2 raises glycosuria and lowers blood glucose levels [31]. Both placebo-controlled and active comparator studies have validated SGLT2i’s ability to lower blood glucose, and its added advantages of lowering blood pressure, promoting weight loss, and low hypoglycemia risk suggest it as a valid second-line treatment for T2DM following metformin [25]. A large number of gΙiflozins entered the game and excited the medical world with their impressive results on cardiorenal protection.

Empagliflozin slows the course of kidney disease, as evidenced by the EMPA-REG outcome trial (Empagliflozin Cardiovascular Outcome Event Trial in Type 2 Diabetes Mellitus Patients), which showed a 39% decrease in deteriorating nephropathy or cardiovascular mortality [32]. Reduced nephropathy has also been demonstrated by the Canagliflozin Cardiovascular Assessment Study (CANVAS) through lower albuminuria progression, decreasing GFR, and decreased need for renal replacement treatment or renal-related mortality [33]. Dapagliflozin, in the Dapagliflozin in Patients with Chronic Kidney Disease trial (DAPA-CKD), reduced the composite risk of ≥50% eGFR decline, kidney failure, or death from renal causes, with benefits seen in both diabetic and non-diabetic CKD (Table 2) [34]. The renoprotective effects of SGLT2i are partly independent of their glucose-lowering action. Proposed mechanisms include lowering intraglomerular pressure through restoration of tubuloglomerular feedback, reducing vascular resistance, promoting natriuresis, and attenuating proximal tubular injury. Given that obesity and hypertension are themselves independent risk factors for diabetic kidney disease, the favorable effects of SGLT2i on body weight and blood pressure likely contribute further to renal benefit [35].

## 8. GLP1-RAs in DN

Glucagon-like peptide type 1 receptor agonists (GLP1-RAs) and dipeptidyl peptidase-4 inhibitors (DPP-4 inhibitors) are examples of incretin-related treatments. The gastrointestinal hormone glucagon-like peptide-1 (GLP1) has a pleiotropic influence on glucose metabolism and functions as an incretin, increasing insulin production. These medications are frequently used as second-line therapy following metformin to reduce hyperglycemia in patients with T2DM. GLP1-RAs directly activate the GLP1 receptor, and DPP-4 inhibitors raise the serum levels of GLP1 by blocking the enzyme that breaks it down. The antihyperglycemic effects of both medication groups are achieved by suppressing glucagon secretion and stimulating insulin secretion [36]. Incretin-based treatments have been shown in several rodent trials to reduce the activity of glomerular leukocyte infiltration, urine indicators of oxidative stress, and biomarkers of inflammation and fibrosis [37,38].

There are many GLP1 mimics on the market right now. By blocking sodium reabsorption by the sodium-hydrogen exchanger-3, GLP1-RA therapy causes a proximal tubular natriuresis in the kidney [39]. The fractional excretion of sodium is therefore markedly increased by GLP1-RA administration, which somewhat mimics the effects of SGLT2 inhibition [40,41]. It is interesting to note that GLP1-RAs do not appear to affect renal blood flow, GFR, or tubuloglomerular feedback, as was observed with SGLT2i [42,43].

In animal models of DN, GLP1-RAs decrease albuminuria and renal morphological abnormalities without influencing renal hemodynamics [44]. Additionally, it has been demonstrated that GLP1-RAs decrease oxidative stress, inflammation, macrophage infiltration, and renal type IV collagen accumulation [45]. A dose-dependent decrease in albuminuria was observed in the SCALE diabetes trial of liraglutide [46]. Liraglutide or semaglutide treatment led to considerable decreases in secondary renal endpoints in the Liraglutide and Renal Outcomes in Type 2 Diabetes trial (LEADER) and Semaglutide and Cardiovascular Outcomes in Patients with Type 2 Diabetes (SUSTAIN-6) trials. This decrease was primarily driven by a decrease in macroalbuminuria progression, but there was no discernible impact on hard outcomes pertaining to kidney function [47,48]. Similarly, there was no significant impact on renal function indices in the Evaluation of Lixisenatide in Acute Coronary Syndrome trial (ELIXA), but there was a substantial decrease in both new onset macroalbuminuria and the deterioration of pre-existing macroalbuminuria [49]. When compared to the sulphonylurea glimepiride, exenatide demonstrated a statistically significant decrease in urine albumin, urinary transforming growth factor β1 (TGFβ1), and type IV collagen in another small randomized clinical trial [50]. While microalbuminuria and ESRD incidence did not significantly change, individuals treated with once-weekly exenatide showed a decrease in new-onset macroalbuminuria as compared to placebo in the Exenatide Study of Cardiovascular Event Lowering Trial trial (EXSCEL) [51]. In T2DM patients with significant kidney dysfunction, the Dulaglutide versus insulin glargine in patients with type 2 diabetes and moderate-to-severe chronic kidney disease (AWARD-7) trial comparing dulaglutide to insulin glargine revealed that both dulaglutide groups exhibited a slower decrease in eGFR over the study period than the glargine group [52]. According to the results of the latest study “Effects of Semaglutide on Chronic Kidney Disease in Patients with Type 2 Diabetes” (FLOW) 1.0 mg once-weekly dose of semaglutide lowered the risk of major kidney and cardiovascular endpoints, and death from any cause [53].

Tirzepatide, a dual glucose-dependent insulinotropic polypeptide (GIP)/GLP1 receptor agonist showed more promising results. In the post hoc analysis of the Effects of tirzepatide versus insulin glargine on kidney outcomes in type 2 diabetes trial (SURPASS-4), tirzepatide prevented kidney function deterioration and albuminuria and halved the risk of the composite endpoint in diabetic patients with high cardiovascular risk compared with insulin glargine [54]. According to the aforementioned findings, GLP1 analogs may help in the regression of DN [4].

## 9. MRAs in DN

Glomerular hypertrophy, sclerosis, and renal fibrosis with decreased renal blood flow are all consequences of mineralocorticoid receptor (MR) overactivation on the kidney, which ultimately leads to renal damage and renal failure [55]. By preventing smooth muscle cell proliferation and decreasing endothelial cell death, finerenone lessens the development of damaged vascular neointima. Finerenone can delay the progression of nephropathy and provide kidney benefits by preventing unfavorable vascular remodeling while restoring vascular integrity and blocking kidney damage caused by MR overactivation [56]. In order to promote endothelium repair and prevent unfavorable vascular remodeling, finerenone inhibits leukocyte recruitment, smooth muscle cell proliferation, endothelial cell death, and the inflammatory response following vascular injury [57].

Cardiorenal outcomes were assessed in two extensive phase III clinical trials, the Cardiovascular Events with Finerenone in Kidney Disease and Type 2 Diabetes trial (FIGARO-DKD) and the Effect of Finerenone on Chronic Kidney Disease Outcomes in Type 2 Diabetes trial (FIDELIO-DKD), which involved patients with T2DM and CKD (Table 2). Finerenone significantly lowered the incidence of renal composite endpoint (death from renal cause, prolonged fall in eGFR ≥ 40% from baseline, or incidence of renal failure) by 23% in the FIGARO-DKD [58,59]. When compared to placebo, finerenone significantly lowered the risk of the renal composite endpoint by 18% in the FIDELIO-DKD [58]. In another brief intervention, finerenone lowered albuminuria in T2DM patients with CKD. It is unknown how it will affect cardiovascular and renal outcomes in the long run. While exhibiting a similar adverse event profile to placebo, finerenone lowers the probability of significant endpoints such as renal failure, a 40% decrease in eGFR, or renal death [57]. In comparison to placebo, finerenone significantly decreased the incidence of renal composite events by up to 23% and UACR by 32%, according to the results of the pooled analysis “Cardiovascular and kidney outcomes with finerenone in patients with type 2 diabetes and chronic kidney disease” (FIDELITY). Finerenone reduced the incidence of all non-lethal kidney outcomes, including ESRD, according to additional research. In all UACR and eGFR phases, finerenone lowers cardiovascular risk in T2DM patients with CKD [60,61]. Comparing finerenone to placebo, the risk of renal composite events was significantly decreased by 29% in patients with established atherosclerotic cardiovascular disease (ASCVD) and by 19% in those without a history of ASCVD. The history of ASCVD had no bearing on the renal benefit of finerenone or its ability to lower all-cause mortality [62].

## 10. Soluble Guanylate Cyclase Activators and DN

The nitric oxide (NO)–soluble guanylate cyclase (sGC)–cyclic guanosine monophosphate (cGMP) pathway plays a central role in vascular regulation and kidney function [63,64,65,66,67,68,69]. sGC is a heterodimeric protein that contains heme and binds to NO, thus playing a significant role in this signaling pathway. When endogenous NO binds to sGC, it activates the enzyme, thus converting guanosine triphosphate into Cyclic guanosine monophosphate (cGMP), serving as a crucial second messenger in signaling and is also involved in the physiological regulation of renal blood flow [65,67]. Additionally, cGMP may exert an antifibrotic effect, as enhanced cGMP signaling can inhibit the formation of extracellular matrix, as well as the production of collagen and fibronectin, and the differentiation of fibroblasts into myoblasts [70]. The stimulation of sGC has been shown to reduce inflammation via the nuclear factor KB−NLRP3 pathway [71]. Diabetes and its common comorbidities, such as CKD, are linked to increased oxidative stress, which in turn decreases NO bioavailability [63,65,72]. This stress can result in the oxidation of sGC and the subsequent loss of heme, impairing the binding of NO to sGC and disrupting NO signaling [72,73]. Consequently, the renoprotective effects of cGMP are hindered, which contributes to the progression of CKD and the acceleration of cardiovascular disease in CKD individuals.

sGC activators represent an option that effectively and selectively activate sGC in conditions of oxidative stress, independently of endogenous NO [63,65,74]. As a result, sGC activators have the potential to restore cGMP signaling during oxidative stress, prevent the progression of CKD, and serve as potential disease-modifying therapies. This hypothesis is corroborated by research conducted in animal models of CKD [75], where sGC activators such as cinaciguat [64,76,77], runcaciguat [67,72,78], and avenciguat [79,80,81] have been shown to reduce proteinuria, morphological kidney damage, and biomarkers of renal injury, irrespective of CKD etiologies. Recently, avenciguat was found to lower UACR in patients with CKD [80]. Last but not least, improvement of atherosclerosis related to diabetes and DN can be achieved by modulating soluble guanylate cyclase [82,83,84,85,86].

## 11. Aldosterone Synthase Inhibitors and CKD

In CKD, elevated levels of aldosterone correlate with heightened proteinuria and albuminuria, a reduction in the eGFR, and an increased likelihood of disease progression [87]. It is believed that aldosterone harms the kidneys by triggering oxidative stress, inflammation, and fibrosis within the glomerular and tubulointerstitial areas [88,89,90]. Treatments that directly inhibit aldosterone production, including aldosterone synthase inhibitors (ASI), are anticipated to more effectively counteract the effects of excess aldosterone compared to RAS inhibitors combined with MRAs. Therefore, AS inhibition signifies a novel potential therapeutic approach for albuminuric CKD and DN [91].

There are limited studies that have assessed the pharmacodynamic and pharmacokinetic characteristics of ASI in individuals with CKD. In a phase 1 trial with parallel groups, patients with different levels of renal function received a single 10 mg dose of baxdrostat, leading to comparable plasma concentration-time profiles and pharmacokinetic metrics in urine across all three cohorts. This indicates that no dose modification is necessary for CKD patients due to pharmacokinetic variations [91]. In another multicenter, randomized, double-blind phase 1–2 trial, 58 diabetic patients with proteinuric CKD were allocated to either BI 690517 (vicadrostat) at doses of 3, 10, or 40 mg, eplerenone 25–50 mg, or a placebo, administered orally once daily for 28 days. Vicadrostat was generally well tolerated, with 13.8% of participants experiencing drug-related adverse events. It significantly lowered plasma aldosterone levels (suppression was noted across all doses of vicadrostat, unlike eplerenone or placebo) and decreased proteinuria [87]. To date, only one randomized, controlled phase 2 trial has been performed with ASI in CKD patients [92]. In this multicenter study, 586 individuals with CKD and proteinuria were enrolled, all of whom were on the maximum tolerated doses of RAAS inhibitors and a serum potassium level < 4.8 mmol/L. According to the study protocol, participants were initially randomized (1:1) during an 8-week phase of empagliflozin or placebo administration. Following this, they were randomized for 14 weeks to vicadrostat once daily at doses of 3, 10, or 20 mg, or a placebo. The primary endpoint was defined as the change in UACR from the second randomization until the conclusion of treatment. Monotherapy with vicadrostat resulted in considerable decline in UACR when compared to placebo, with the reduction being dose-dependent. The combination of vicadrostat with empagliflozin produced similar reductions in UACR, suggesting that ASIs and SGLT2is may work through complementary mechanisms to lower renal risk. In terms of safety, hyperkalemia was observed in 10%, 15%, and 20% of participants receiving 3 mg, 10 mg, and 20 mg of vicadrostat, respectively, compared to 6% in the placebo group (with or without empagliflozin). Nevertheless, 86% of the hyperkalemia cases did not necessitate any intervention. Adrenal insufficiency was noted as a special interest adverse event in 2% of patients treated with vicadrostat, in contrast to 1% among those receiving placebo, while no deaths related to treatment were reported throughout the study [92].

## 12. Combination Therapy in DN

Treating DN and retarding its progression is a complex skill that requires a combination of drugs acting concomitantly. Dapagliflozin and telmisartan combination effectively improves kidney function recovery, controls UACR, and reduces inflammatory responses in DN patients [6]. In the “Finerenone with Empagliflozin in Chronic Kidney Disease and Type 2 Diabetes” (CONFIDENCE) clinical trial in individuals suffering from both CKD and T2DM, the initial treatment combining finerenone and empagliflozin resulted in a more significant decrease in UACR compared to either medication used separately [93]. Notably, a greater than 30% reduction in UACR was observed within 14 days of co-initiation of both therapies—a threshold recommended to slow the progression of kidney disease in patients with CKD [93]. Currently, numerous new potential therapeutic agents have been created, and several of these have undergone clinical trials [86].

In the Randomized Clinical Trial on the Effect of Bardoxolone Methyl on GFR in Diabetic Kidney Disease Patients (TSUBAKI) study, the nuclear factor erythroid 2-related factor 2 activator, bardoxolone methyl, demonstrated an enhancement in the GFR among patients suffering from DKD [94]. Along those lines, novel agents targeting oxidative stress and inflammation pathways have garnered significant attention, including pentoxifylline, inhibitors of apoptosis signal-regulating kinase-1, C-C chemokine receptor 2 inhibitors, and Janus kinase-1/2 inhibitors [86].

Moreover, endothelin-1 receptor A antagonists along with soluble guanylate cyclase stimulators are anticipated to influence renal hemodynamics. In the “Atrasentan and renal events in patients with type 2 diabetes and chronic kidney disease” trial (SONAR), participants who responded to atrasentan, an antagonist exhibiting significant efficacy and selectivity for the ET-A receptor, experienced a decrease in albuminuria without any fluid retention during the initial 6-week open-label phase were enrolled and subsequently randomized. Over a median follow-up period of 2.2 years, atrasentan markedly reduced the risk of serum creatinine doubling and ESRD in comparison to placebo [95]. Additionally, certain preclinical investigations indicate that hypoxia-inducible factor prolyl hydroxylase inhibitors, which modulate various inflammatory and oxidative stress pathways, may lead to a reduction in albuminuria in DKD [96,97]. Furthermore, advanced glycation end-product inhibitors and epigenetic-related treatments have shown potential as viable therapeutic options for DKD in preclinical research [86]. The identification of new therapeutic targets may offer innovative treatment avenues for addressing DKD.

Understanding the pathophysiology of kidney disease, along with early recognition and initiation of currently available therapies, remains critical to slowing its progression. Treatment with newer agents has been shown to reduce albuminuria. The key question is whether potentially greater benefits could be achieved through early and intensive combination therapy, and whether such an approach could more effectively slow disease progression. Following the use of RAS blockers, treatment may be continued with the co-initiation of a SGLT2-i, with an ns-MRA if eGFR ≥ 20 mL/min/1.73 m^2^ (continued until dialysis or transplantation), and/or with a GLP-1 RA if obesity is a significant comorbidity. These agents have recently been added to the therapeutic arsenal for DKD. The recently published results from the CONFIDENCE clinical trial reinforce this perspective.

**Figure 2:**
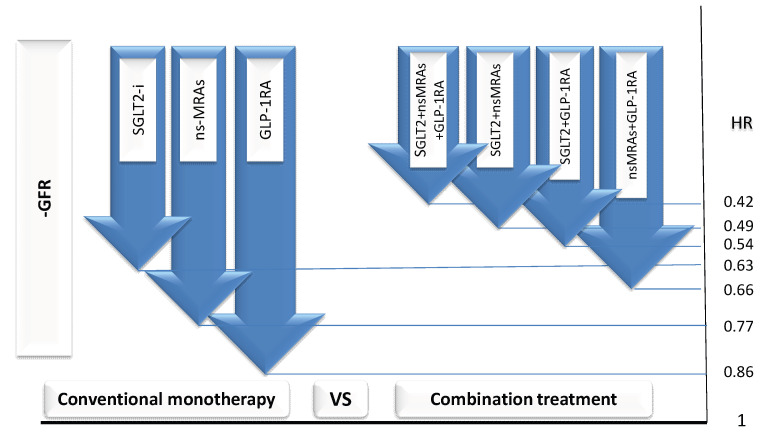
Comparative impact of conventional monotherapy versus early intensive combination therapy on the rate of estimated glomerular filtration rate (eGFR) decline in diabetic kidney disease. Large downward arrows represent the magnitude of eGFR decline over time. Conventional monotherapy (**left**) shows greater decline compared with combination regimens (**right**), which include sodium–glucose cotransporter 2 inhibitors (SGLT2-i), non-steroidal mineralocorticoid receptor antagonists (ns-MRAs), and glucagon-like peptide-1 receptor agonists (GLP-1 RAs). Combination therapies demonstrate lower hazard ratios (HRs) for disease progression, as indicated on the right axis, with triple regimens showing the greatest preservation of renal function.

Evidence suggests that the progressive decline in renal function is addressed more effectively by early and intensive combination therapy compared with conventional monotherapy (Figure 2) [98]. The next step may involve triple combination therapy—adding a GLP-1 RA to the initial combination and an ns-MRA to the alternative combination.

## 13. Limitations for the Combination Treatment

However, there are important limitations to the aforementioned treatment strategy. In patients with kidney disease, hyperkalemia is a significant limitation to the administration of finerenone in combination therapy and must be corrected prior to initiation. In addition, very low kidney function (e.g., eGFR 20–25 mL/min/1.73 m^2^) remains a barrier to the administration of SGLT2-i and/or finerenone. It is also well recognized that not all patients respond uniformly to treatment, underscoring the importance of the histopathological evaluation of renal biopsy specimens in DKD. As a rule, earlier and more intensive combination therapy with these newer agents is expected to yield better outcomes. Persistence with therapy can help overcome both medical inertia and patient non-adherence, which remain significant challenges in this population. Finally, despite encouraging results, a notable limitation is the absence of long-term studies evaluating all of these newer therapeutic approaches.

## 14. Conclusions

The management of diabetic nephropathy has entered a transformative era, driven by advances in pharmacotherapy and a deeper understanding of its pathophysiological mechanisms. While complete regression of kidney damage remains a challenging goal, accumulating evidence suggests that early intervention—particularly with individualized combinations of RAAS inhibitors, SGLT2i, GLP1-RAs, MRAs—can significantly slow progression and, in select cases, induce partial regression.

## Figures and Tables

**Table 1:** Classification of glomerular, interstitial, and vascular lesions of diabetic nephropathy.

**Glomerular**					**Interstitial and Vascular**				
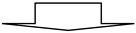					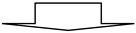				
**I**	**IIa**	**IIb**	**III**	**IV**	Interstitial lesions.	Interstitial inflammation.	Vascular lesions arteriolar hyalinosis.	Presence of large vessels.	Arteriosclerosis (score worst artery).
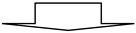					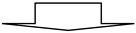				
Mild or nonspecific LM changes and EM-proven GBM thickening.	Mild mesangial expansion.	Severe mesangial expansion.	Nodular sclerosis (Kimmelstiel– Wilson lesion).	Advanced diabetic glomerulosclerosis.	No IFTA 25% 25% to 50% 50%	Absent 0. Infiltration only in relation to IFTA 1. Infiltration in areas without IFTA 2.	Absent 0. At least one area of arteriolar hyalinosis 1. More than one area of arteriolar hyalinosis 2.	Yes/no	No intimal thickening 0. Intimal thickening less than thickness of media 1. Intimal thickening greater than thickness of media 2.
Biopsy does not meet any of the criteria mentioned below for class II, III, or IV. GBM 395 nm in female and 430 nm in male individuals 9 years of age and older.	Biopsy does not meet criteria for class III or IV. Mild mesangial expansion in 25% of the observed mesangium.	Biopsy does not meet criteria for class III or IV. Severe mesangial expansion in 25% of the observed mesangium.	Biopsy does not meet criteria for class IV. At least one convincing Kimmelstiel– Wilson lesion.	Global glomerular sclerosis in 50% of glomeruli. Lesions from classes I through III.					

LM, light microscopy. IFTA, Interstitial fibrosis and tubular atrophy. On the basis of direct measurement of GBM width by EM, these individual cutoff levels may be considered indicative when other GBM measurements are used [13]. Mesangial expansion is substantially correlated with GFR, albuminuria, and hypertension, although glomerular basement membrane width is somewhat less strongly correlated [8,9].

**Table 2:** Therapeutic approaches in halting diabetic kidney disease progression.

	Study	Subjects in the Study	Treatment	Outcome	Results
RAASi	IDNT	1715 hypertensive patients with nephropathy due to type 2 diabetes.	Irbesartan (300 mg daily), amlodipine (10 mg daily) vs. placebo.	Doubling of baseline serum creatinine concentration, the development of ESRD, or death from any cause.	Doubling of serum creatinine concentration was 33 percent lower in the irbesartan group than in the placebo group (*p* = 0.003) and 37 percent lower in the irbesartan group compared to amlodipine (*p* < 0.001).
	RENAAL	1513 hypertensive patients.	Losartan (50 to 100 mg once daily) vs. placebo, both taken in addition to conventional antihypertensive treatment.	Doubling of the baseline serum creatinine concentration, ESRD, or death.	Losartan reduced the incidence of a doubling of the serum creatinine concentration (risk reduction, 25% *p* = 0.006) and ESRD (risk reduction, 28% *p* = 0.002) but had no effect on mortality.
SGLT2 i	EMPA-REG	7020 patients with type 2 diabetes at high cardiovascular (CV) risk.	Empagliflozin (10 mg or 25 mg) vs. placebo.	Death from cardiovascular causes, nonfatal myocardial infarction, or nonfatal stroke.	Lower risk of cardiovascular death (3.7% vs. 5.9% in the placebo group; 38% relative risk reduction), hospitalization for HF (2.7% and 4.1%, respectively; 35% relative risk reduction), and all-cause mortality in empagliflozin-treated patients.
	CANVAS	10,142 participants with type 2 DM and high CV risk.	Canagliflozin vs. placebo.	Death from cardiovascular causes, nonfatal myocardial infarction, or nonfatal stroke.	The rate of the primary outcome was lower with canagliflozin than with placebo (hazard ratio, 0.86; 95% confidence interval [CI], 0.75 to 0.97; *p* < 0.001 for noninferiority; *p* = 0.02 for superiority). The results showed a possible benefit of canagliflozin with respect to the progression of albuminuria (hazard ratio, 0.73; 95% CI, 0.67 to 0.79) and the composite (hazard ratio, 0.60; 95% CI, 0.47 to 0.77).
	DAPA –CKD	4304 participants with and without DM and high CV risk.	Dapagliflozin vs. placebo.	Rate of kidney function deterioration.	Lower decline in the eGFR of at least 50%, ESRD, or mortality.
GLP1-RAs	LEADER	9340 patients of high CV risk.	Liraglutide vs. placebo.	Rate of kidney function deterioration.	26% reduction of the de novo macroalbuminuria; 19% reduction of UACR.
	SUSTAIN-6	3297 patients with T2DM and CVD or with CV risk factors.	Semaglutide vs. placebo.	Rate of kidney function deterioration.	New or worsening nephropathy occurred less frequently; HR = 0.64 (0.46–0.88), *p* = 0.005.
	ELIXA	6068 patients with T2DM and acute coronary syndrome.	Lixisenatide vs. placebo.	Progression of albuminuria.	Lixenatide reduces progression of UACR in macroalbuminuric patients.
	EXSCEL	14,752 patients (73% had CVD).	Extended-release exenatide vs. placebo.	Rate of kidney function deterioration.	Reduction of eGFR with 40% decline, RRT or new macroalbumiuria; HR = 0.85 (0.73–0.98, *p* = 0.027).
	AWARD-7	577 patients with T2DM and advanced CKD.	Dulaglutide vs. insulin glargine.	eGFR and UACR change from baseline.	Dulaglutide reduced eGFR decline compared to insulin glargine.
	FLOW	3533 patients with T2DM and CKD.	Semaglutide vs. placebo.	Major kidney disease events, a composite of the onset of kidney failure, at least a 50% reduction in eGFR from baseline or death from renal or CV causes.	24% lower risk of a primary-outcome event in the Semaglutide group. The results for all confirmatory secondary outcomes favored Semaglutide: mean annual eGFR slope was less steep, the risk of major CV events was 18% lower (HR = 0.82, 0.68–0.98; *p* = 0.029), and the risk of all-cause mortality was 20% lower (HR = 0.80; 0.67–0.95, *p* = 0.01).
Ns-MRAs	FIGARO-DKD	7437 patients with CKD and type 2 DM	Finerenone vs. placebo.	Death from CV causes, nonfatal myocardial infarction, nonfatal stroke, or hospitalization for heart failure. The first secondary outcome was a composite of renal failure, a sustained decrease from baseline of at least 40% in eGFR, or renal death.	A primary outcome event occurred in 12.4% of the finerenone group and in 14.2% of the placebo group (hazard ratio, 0.87; 95% confidence interval [CI], 0.76 to 0.98; *p* = 0.03), with the benefit driven primarily by a lower incidence of hospitalization for heart failure (hazard ratio, 0.71; 95% CI, 0.56 to 0.90). The secondary composite outcome occurred in 9.5% of the finerenone and in 10.8% of the placebo group (hazard ratio, 0.87; 95% CI, 0.76 to 1.01).
	FIDELIO-DKD	5734 patients with CKD and type 2 DM.	Finerenone vs. placebo.	Kidney failure, a sustained decrease of at least 40% in the eGFR from baseline, or death from renal causes.	A primary outcome occurred in 17.8% of the finerenone and 21.1% of the placebo group (hazard ratio, 0.82; 95% confidence interval [CI], 0.73 to 0.93; *p* = 0.001).
	FIDELITY	13,026 patients combining data from FIGARO-DKD and FIDELIO-DKD.	Finerenone vs. placebo.	A composite of CV death, nonfatal myocardial infarction, nonfatal stroke, or hospitalization for heart failure, and a composite of kidney failure, a sustained ≥57% decrease in estimated glomerular filtration rate from baseline over ≥4 weeks, or renal death.	The composite cardiovascular outcome occurred in 825 (12.7%) patients receiving finerenone and 939 (14.4%) receiving placebo (hazard ratio (HR), 0.86; 95% confidence interval (CI), 0.78–0.95; *p* = 0.0018). The composite kidney outcome occurred in 5.5% of patients receiving finerenone and 7.1% of patients receiving placebo (HR, 0.77; 95% CI, 0.67–0.88; *p* = 0.0002).

RAASi: renin- angiotensin aldosterone inhibitors, SGLT2i: sodium-glucose cotransporter 2 inhibitors, GLP1-RAs: glucagon-like peptide-1 receptor agonists, ns-MRAs: non-steroidal mineralocorticoid receptor antagonists.
